# Sirolimus Suppresses Phosphorylation of Cofilin and Reduces Interstitial Septal Thickness in Sporadic Lymphangioleiomyomatosis

**DOI:** 10.3390/ijms22168564

**Published:** 2021-08-09

**Authors:** Yen-Lin Huang, Po-Ru Chen, Ying-Ju Lai, Hsao-Hsun Hsu

**Affiliations:** 1 Department of Pathology, National Taiwan University Hospital, National Taiwan University College of Medicine, Taipei 10002, Taiwan; fdflame2@gmail.com; 2 Department of Pathology, National Taiwan University Cancer Center, National Taiwan University College of Medicine, Taipei 106328, Taiwan; 3 Graduate Institute of Clinical Medical Sciences, Chang Gung University College of Medicine, Taoyuan 33353, Taiwan; M0800301@cgu.edu.tw; 4 Department of Respiratory Therapy, Chang Gung University College of Medicine, Taoyuan 33353, Taiwan; 5 Cardiovascular Division, Chang Gung Memorial Hospital, Taoyuan 33353, Taiwan; 6 Department of Respiratory Care, Chang-Gung University of Science and Technology, Chiayi 61363, Taiwan; 7 Division of Thoracic Surgery, Department of Surgery, National Taiwan University Hospital and National Taiwan University College of Medicine, Taipei 10002, Taiwan

**Keywords:** lymphangioleiomyomatosis, sirolimus, p-cofilin, interstitial septal thickness, mTOR

## Abstract

Sporadic lymphangioleiomyomatosis (S-LAM) is a rare lung disease characterized by the proliferation of smooth muscle-like LAM cells and progressive cystic destruction. Sirolimus, a mammalian target of rapamycin (mTOR) inhibitor, has a proven efficacy in patients with LAM. However, the therapeutic mechanisms of sirolimus in LAM remain unclear. We aimed to evaluate sirolimus-related lung parenchymal changes and the potential effect in LAM cells and modulating pathological cystic destruction. Lung specimens were examined for histopathological changes by HMB45 staining and compared the LAM patients treated with and without sirolimus. We detected the overexpression of mTOR, HMB45, and phosphorylation of cofilin (p-cofilin) in LAM patients. Sirolimus showed efficacy in patients with LAM, who exhibited a reduced expression of mTOR and p-cofilin as well as reduced interstitial septal thickness. In addition, sirolimus suppresses mTOR and p-cofilin, thus suppressing the migration and proliferation of LAM cells isolated from the patient’s lung tissue. This study demonstrates that interstitial septal thickness, as determined by histological structural analysis. Sirolimus effectively reduced the expression of p-cofilin and interstitial septal thickness, which may be a novel mechanism by sirolimus. Moreover, we develop a new method to isolate and culture the LAM cell, which can test the possibility of medication in vitro and impact this current study has on the LAM field. The development of approaches to interfere with mTOR-cofilin1-actin signaling may result in an option for S-LAM therapy.

## 1. Introduction

Lymphangioleiomyomatosis (LAM) is a rare progressive and systemic disease that predominantly affects young women of reproductive age [1]. In the lung, LAM is characterized by the proliferation or patchy aggregates of bland-appearing smooth muscle-like LAM cells throughout the interstitium; these cells destruct the alveolar wall and form thin-walled cysts of variable size, leading to loss of lung function [2]. LAM cells also frequently migrate and infiltrate into blood vessels or lymphatic channels, resulting in recurrent pneumothorax and chylous effusions [3]. LAM can also involve other organs in rare cases, including the pancreas, retroperitoneum, pelvis, mediastinum, kidney, uterus and mesentery [4].

Genetically, LAM occurs in a sporadic form (sporadic LAM) or association with tuberous sclerosis complex (TSC-LAM). Hamartin (TSC1) and tuberin (TSC2) are tumor suppressor genes encoding proteins that negatively regulate cell growth in a variety of systems [5]. Both forms of LAM are caused by loss-of-function mutations in the TSC1 or TSC2 gene. In addition, circulating LAM cells are heterogeneous with neoplastic, metastatic, and cancer-stem cell-like properties [6]. The encoded hamartin and tuberin proteins regulate the intracellular serine/threonine kinase mTOR signaling pathway, a significant regulator of cell size, migration, proliferation, and survival [7,8,9]. The TSC1-TSC2 complex, a tumor suppressor, forms when TSC1 and TSC2 bind via their coiled-coil domains to form an intracellular complex, which helps switch the protein Ras homolog enriched in brain (Rheb) from its active state, Rheb-GTP, to its inactive state [10]. Rheb is ubiquitously expressed in humans and is an activator in the mammalian target of rapamycin complex 1 (mTORC1) pathway; the activation of mTORC1, a serine/threonine kinase, leads to phosphorylation cascades that result in cell growth and proliferation [11]. mTORC2 promotes actin polymerization in cells by activating the Rho-GTPase (Rac1)-p21-activated kinase-1 (PKA)-cofilin pathway to enhance the activity of the actin depolymerase cofilin [8,12,13].

Sirolimus (rapamycin), an mTOR inhibitor, has shown efficacy in stabilizing lung function, reducing serum vascular endothelial growth factor-D (VEGF-D) levels, resolving chylous effusions and improving 6-min walking distances and is associated with an improved quality of life in patients with LAM [14,15,16,17]. Although emerging evidence has revealed the efficacy of sirolimus in LAM disease, some patients treated with sirolimus still progress, and only lung transplantation (LT) remains a standard treatment option for patients with advanced-stage LAM [18]. The inhibition of the Src/Akt/mTOR/cofilin pathway impaired the organization of actin cytoskeleton and regulated invasion, intravasation, and metastasis of mammary tumors [19,20,21]. The fine balance between the expression and subcellular localization of cofilin-1 (phosphorylated or non-phosphorylated) and its regulator LIMKs is crucial for changes in the dynamics of the actin cytoskeleton [22] to regulated the proliferation and migration process of tumor cells [19,20,23].

Aside from lung function tests and chest computed tomography (CT), no other tools have been used to follow and evaluate the severity of pulmonary LAM. In addition, rare research has studied the histopathological changes in the lung after sirolimus therapy.

Our study aimed to report the clinical characteristics of patients with advanced LAM in the Taiwanese population. Furthermore, we used surgical specimens to thoroughly investigate the morphological changes associated with pulmonary LAM and search for possible pathological parameters corresponding to clinical outcomes. Then, we established a method to isolate the LAM cell from lung tissue of LAM patients to investigate the in vitro mechanism of sirolimus, the mTOR inhibitor, in LAM cells. By comparing patients treated with and without sirolimus, we demonstrated for the first time the morphological changes associated with sirolimus treatment. We provided direct evidence of sirolimus efficacy from a pathological perspective and the inhibition of the mTOR/p-cofilin pathway to suppress migration and proliferation in LAM cells.

## 2. Results

### 2.1. Pathological and Histological Analysis of LAM Patient Lung Tissues

**Figure 1:**
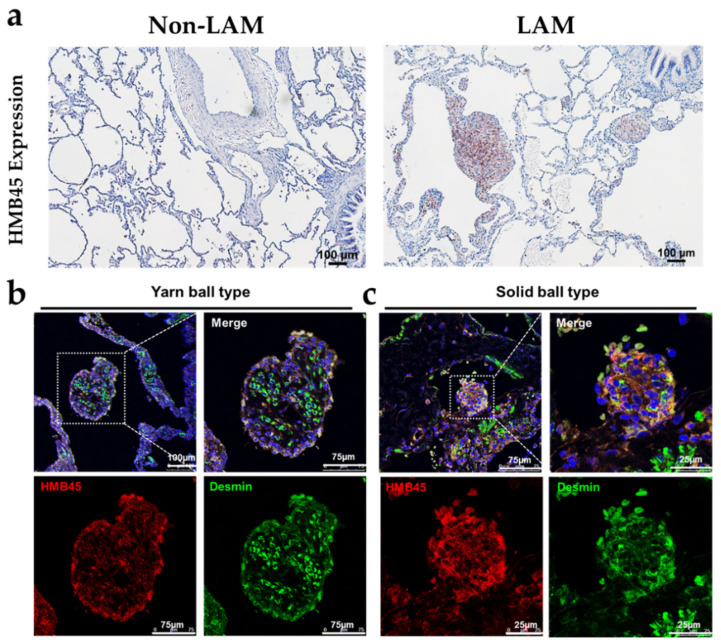
Histological appearance of lymphangioleiomyomatosis (LAM). (**a**) Immunohistochemistry shows HMB45 expression in cysts with cystic walls of variable thickness (scale bar: 100 μm). The HMB45-positive LAM cells aggregated to form a solid mural nodule on the cyst wall, identified by brown staining. In contrast, anti-HMB45 signals were hardly detectable in the lungs of non-LAM patients. (**b**) Immunohistochemistry shows HMB45 expression in the external layer of the LAM nodule, with desmin double staining of SMC-like cells (scale bar: 75 μm). Yarn ball: many SMC-like cells wrap around the LAM cell fascicles, producing anastomosing capillary-like channels reminiscent of a yarn ball. (**c**) Representative HMB45- and desmin-positive LAM cells aggregate to form a solid ball nodule (scale bar: 25 μm).

LAM is a sporadic disease of the lungs and lymphatic system associated with tuberous sclerosis and exclusively affects females, developing before menopause [1]. Cyst formation with variable cystic wall thickness and LAM cells are detectable by HMB45 positivity [24]. Frequently, the HMB45-positive LAM cells aggregated to form a mural nodule on the cyst wall or protruding into the cystic spaces, exhibiting a ball-like architecture (Figure 1a right). In contrast, anti-HMB45 signals are hardly detectable in the lungs of non-LAM (Figure 1a left). In the lung tissues of patients with LAM, we investigated the expression levels of HMB45, desmin and changes in cyst formation. The immunofluorescence analysis revealed abundant anti-HMB45 signals in the cystic walls of patients with LAM. These signals colocalized with the anti-desmin signal, indicating that HMB45 was expressed in SMC-like LAM cells. To further investigate the nodules or ball-like structures, we performed double staining for desmin (green) and HMB45 (red) via immunofluorescence to identify the two main structures (Figure 1b,c). One form was determined to be LAM cell bundles separated by delicate capillary-like cannels resembling a yarn ball (Figure 1b). In contrast, the other was determined to be solid-type nodules composed of pure interlacing fascicles of LAM cells, which may have resulted from a tangential cut of the thick cystic wall (Figure 1c). These findings showed that nodule-like structures were characterized by HMB45 positivity and two main architectures in pulmonary LAM.

### 2.2. Sirolimus Suppresses mTOR Expression in Lung Tissue of Patients with LAM

**Figure 2:**
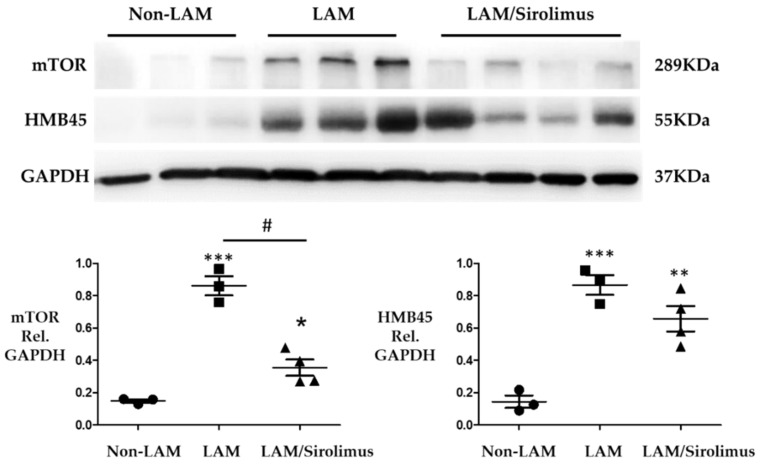
Effect of sirolimus on decreasing mTOR expression. Representative quantifications of the immunoblot densitometries of protein expression are shown for lungs from the two groups. mTOR (289-kDa band) expression was increased in the lung tissues of LAM patients, and this change was reversed by administering sirolimus (LAM/sirolimus). HMB45 (55-kDa band) expression was increased in the lung tissues of LAM patients but did not decrease significantly after sirolimus administration (LAM/sirolimus), relative (Rel.). The bars represent the mean ±SEM for *n* = 3-4 samples. * *p* < 0.05, ** *p* < 0.01, *** *p* < 0.001 compared with the non-LAM group. # *p* < 0.05 compared with the LAM group without sirolimus treatment.

Sirolimus has been shown to affect the mTOR signaling pathway [25,26] and HMB45 is a marker for both sporadic LAM and TSC-LAM [23,24,27,28]. The experiments in this study were designed to investigate whether sirolimus plays a role in suppressing the expression of mTOR and HMB45. We obtained lung tissues from three non-LAM patients, three LAM patients treated without sirolimus, and four LAM patients treated with sirolimus to further evaluate the effect of sirolimus, a mTOR inhibitor. Western blotting revealed that the lung tissues from LAM patients exhibited higher mTOR and HMB45 levels than those from non-LAM patients (Figure 2). Sirolimus treatment of patients with LAM reduced mTOR expression (Figure 2) but did not significantly change the HMB45 levels. These findings confirmed that sirolimus functions as an mTOR inhibitor in LAM lung tissue.

### 2.3. Interstitial Septal Thickness Is a Marker for the Efficacy of Sirolimus in LAM

**Figure 3:**
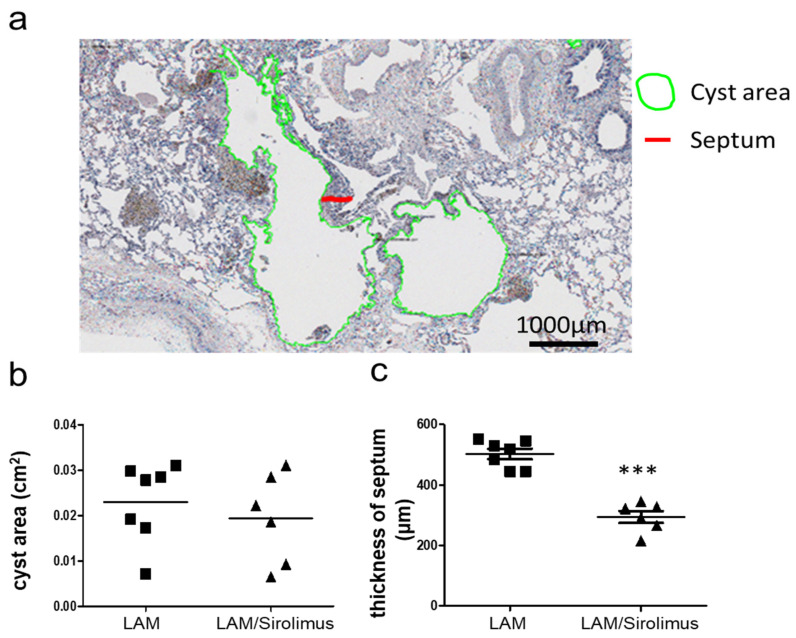
The effect of sirolimus on the HMB45-stained LAM tissue structure via histological analysis. (**a**) The cyst area (green circle) and thickness of the septum (red line) as revealed by HMB45 staining and pathological study of lung tissues from patients with LAM. Scale bar: 1000 μm. Quantitative analysis of the (**b**) cyst area and (**c**) septum thickness in patients with LAM treated without and with sirolimus. HMB45 staining was labeled the LAM cells to defined the size of cyst area and thickness of the LAM septa. At least 3–5 slides were evaluated from every patient. We have 7 LAM patients (total 188 septa counts, 93 cyst counts) vs. 6 LAM patients with sirolimus (total 128 septa counts, 72 cyst counts). The data are presented as the mean±SEM (*n* = 6–7); *** *p* < 0.001.

The effect and mechanism of sirolimus in human LAM cells are still unclear. To investigate the histopathological changes in the lung after sirolimus therapy, we established measurement models to observe the LAM cyst area and interstitial septal thickness in the lung tissues of patients with LAM treated with or without sirolimus by immunohistochemically staining for HMB45, a LAM cell marker (Figure 3a). Histological analysis and measurement of HMB45 staining in the lung tissue sections demonstrated that the LAM interstitial septum was significantly thicker in patients with LAM left untreated than in those treated with sirolimus (Figure 3b) and that the LAM cystic area was not changed significantly (Figure 3c), suggesting that sirolimus treatment prevents interstitial LAM septal remodeling and thickening. LAM occurs in two settings TSC-LAM in women with tuberous sclerosis complex, 30% to 40% of women with TSC mutation and disease in other organs, especially skin [29,30]. Sporadic LAM in women who do not have TSC, a non-inherited form of LAM disease, exclusively affects females, developing before menopause. The lung cysts of TSC-LAM are often mild, but S-LAM is often profuse and lung function worsens in the disease progression [30]. The patients included in our cohort are all sporadic LAM. Due to the low prevalence of tuberous sclerosis complex in Taiwan, we do not check the genetic screening for TSC1 or TSC2 mutation. As to clinical diagnostic criteria [29], it was agreed that an individual who has LAM without TSC features in other organs, especially skin, does not meet criteria for a definite diagnosis of TSC, and usually diagnosed as sporadic LAM. The patients in our study all received lung transplant. Before surgery, an image study including sonography and computed tomography is performed to examine if there were any abnormal findings in other organs. A well-structured multidisciplinary discussion (MDD) on the etiological assessment, diagnosis, and management of each LAM patient is routinely performed. If there is a chance of TSC-LAM, the patient is referred to other subspecialties for further treatment. The committee of lung transplant in Taiwan Ministry of Health and Welfare also check if these patients are sporadic LAM before surgery. The clinical, and physiological characteristics of the patients with S-LAM in two groups are summarized in Table 1.

### 2.4. The Effect of Sirolimus on p-Cofilin in the Interstitial LAM Septum

**Figure 4:**
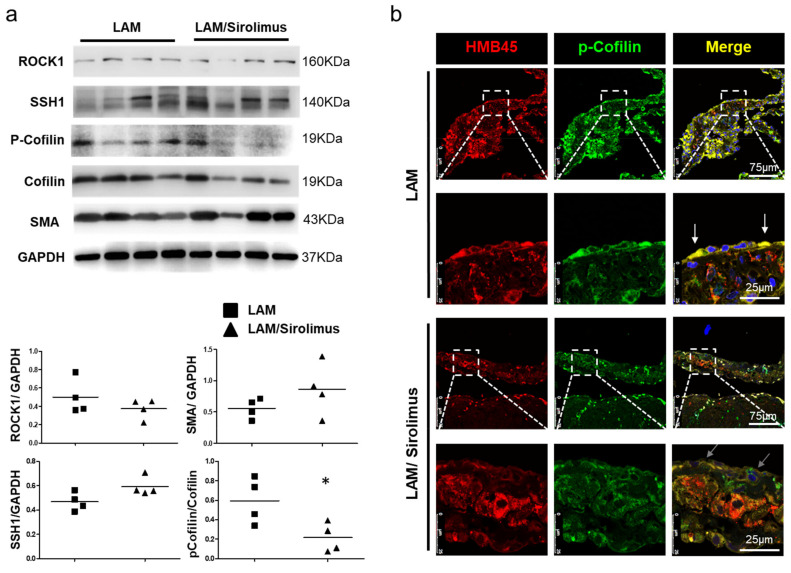
Sirolimus inhibits p-cofilin expression in the LAM septum. (**a**) A representative Western blot is shown above, and quantitative data are shown below to demonstrate the ROCK, SSH1, LIMK, and p-cofilin/cofilin protein levels in human lung tissue. The ratio of p-cofilin to cofilin indicated decreased cofilin phosphorylation in the lung tissues of LAM patients treated with sirolimus compared with untreated patients. Relative protein expression values normalized to GAPDH (*n* = 4) or cofilin. The data are presented as the mean±SEM; * *p* < 0.05 versus the LAM group. (**b**) Immunofluorescence showing the localization of *p*-cofilin and HMB45 in the LAM septum. Scale bar: 75 and 25 μm. The phosphorylation level of cofilin was decreased specifically at the edge of the LAM interstitial septum (white arrows).

mTOR has been reported to regulate the LIM kinase (LIMK) and cofilin signaling pathways [31]. The phosphorylation of cofilin, an actin-regulating protein, determines the direction of cell migration [32]. To determine whether sirolimus regulates LAM interstitial septal thickness and is associated with the mTOR signaling cascade, we evaluated the expression of the mTOR signaling-related proteins ROCK1, LIMK, Slingshot-1 (SSh1), and cofilin in LAM patients treated with or without sirolimus. Further Western blotting revealed that sirolimus reduced the phosphorylation of cofilin in the lung tissue lysate. Research on the underlying molecular mechanism showed that sirolimus suppressed the activation of the mTOR pathway. In this study, evaluations of histopathological changes showed that sirolimus regulated the interstitial septal thickness of human LAM cells. A specific inhibitor of mTOR, sirolimus, was employed to further investigate cofilin, and the Western blotting results revealed lower p-cofilin levels in the lung tissues of LAM patients treated with sirolimus than in LAM patients not treated with the inhibitor, and ROCK1, LIMK, and SSh1 expression did not change significantly (Figure 4a). The phosphorylation level of cofilin decreased specifically at the edge of the interstitial LAM septum (Figure 4b). Together, these data suggest that the inhibitory effect of sirolimus on mTOR-stimulated LAM interstitial septal thickness might be prevented by cofilin phosphorylation. These results indicate that sirolimus may regulate the interstitial septal thickness of human LAM cells via the mTOR/cofilin pathway. In addition, the development of approaches to interfere with mTOR-cofilin1-actin signaling may result in interesting options for S-LAM therapy.

### 2.5. Sirolimus, an mTOR Inhibitor, Downregulates p-Cofilin Expression in LAM Cells

**Figure 5:**
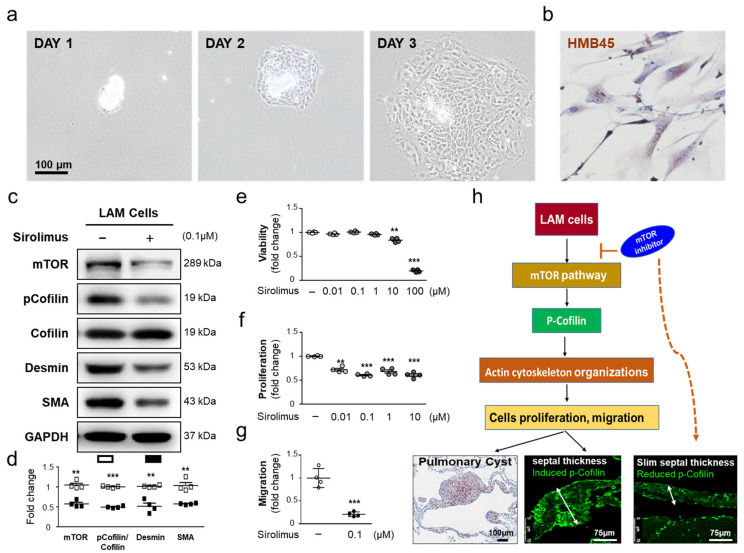
Effect of sirolimus on LAM cell proliferation and migration associated with mTOR and p-cofilin expression. (**a**) The LAM cell clusters (size between 70 μm to 100 μm) was isolated from LAM patients, the phase contrast images show the morphology of LAM cells in adherent culture at day 1, day 2, and day 3. The images were obtained using 40× objectives. (**b**) The primary culture LAM cells were identified by the specific markers HMB45 to characterize the LAM cells. Immunocytochemistry showing HMB45 localization (brown staining; nuclear staining with hematoxylin in blue). (**c**) LAM cells were treated with or without sirolimus (0.1 μM). Western blot was used to assess the protein expression levels in whole-cell extracts. (**d**) Relative expression values of mTOR, pCofilin, tCofilin, desmin, and SMA obtained by densitometry. The data are presented as the mean ±SEM of three samples in each group. ** *p* < 0.01 and *** *p* < 0.001 compared with LAM cells without sirolimus. (**e**) cell viability, (**f**) Sirolimus treatment inhibited the proliferation of LAM cells. (**g**) Sirolimus treatment inhibited the migration of LAM cells. The data (mean ±SEM [*n* = 4]) are presented as the fold change in cell numbers compared with those in the untreated group. ***p < 0.001 compared with the untreated group. (**h**) the schema: possible mechanisms of mTOR inhibitor in LAM via decreased p-cofilin.

Histological data showed that the inhibitory effect of sirolimus on mTOR-stimulated LAM interstitial septal thickness might be prevented by cofilin phosphorylation. Sirolimus may regulate the interstitial septal thickness of human LAM cells via the mTOR/cofilin pathway. To further understand the effects of sirolimus on the mTOR signaling pathway and p-cofilin, we isolated the LAM cells from LAM cell clusters (size between 100 μm to 70 μm) of LAM patients’ lung tissue (Figure 5a) and identified by positive HMB45 expression (Figure 5b). We hypothesized that sirolimus, an mTOR inhibitor, may suppress the expression of mTOR and p-cofilin to affect the cell proliferation and migration ability. Western blot analysis showed that mTOR, p-Cofilin, smooth muscle-Actin (SMA) protein, and desmin expression was decreased following sirolimus treatment at 0.1 μM (Figure 5c,d), suggesting that sirolimus downregulated these proteins in LAM cell. As mTOR and p-cofilin play an important role in the migration of cells and cancer cell metastasis [32,33,34], we exposed LAM cell to sirolimus to investigate its effect on cell viability, proliferation, and migration. Treatment of LAM cells with sirolimus (0.1 μM) led to a significant decrease in serum-induced proliferation and migration (Figure 5e–g). To the best of our knowledge, the method for isolating LAM cells and culture is the first to develop in the human cell model to exhibit different behaviors in response to sirolimus. Taken together, these findings indicated that sirolimus decreases mTOR, p-cofilin, SMA protein, and desmin and may subsequently facilitate the proliferation and migration of LAM cells (Figure 5h).

## 3. Discussion

The significant outcomes of our study were to perform histological structural analysis in LAM patients treated with and without sirolimus. In addition, the interstitial septal thickness may be a diagnostic parameter for LAM patients. It may reveal the biological difference between LAM patients treated with and without sirolimus in a Taiwanese population. In addition, sirolimus effectively reduced the expression of p-cofilin and interstitial septal thickness, which may be a novel mechanism by sirolimus. Moreover, we have developed a new method from our cardiopulmonary research laboratory to isolate and culture the LAM cell, which is able to test the possibility of medication in vitro and impact this current study has on the LAM field.

Several studies have shown that sirolimus stabilizes lung function and improves the quality of life for patients with LAM [4,14]. By utilizing LT specimens, we studied sirolimus-related lung parenchymal changes via histological and molecular analyses of the lungs of patients with LAM. The size and number of cysts were not significantly associated with sirolimus therapy in our cohort, concurring with a previous study revealing that the cyst volume increased at a slow rate and did not decrease during sirolimus therapy, as evaluated using high-resolution computed tomography (HRCT) scans [15]. Nonetheless, the LAM cell-containing septum is significantly thicker in patients not receiving sirolimus therapy, and this parameter is not detectable by HRCT and has therefore not been described previously.

Sporadic lymphangioleiomyomatosis (LAM) is a rare lung disease characterized by the proliferation of smooth muscle-like LAM cells with positive reactivity with monoclonal antibody HMB45 and progressive cystic destruction. The research limitations to establish a human cell model, are most related to the rarity of LAM disease and the limited availability of fresh lung tissue samples. In addition, the LAM cells are difficult to isolate, and lose differentiated features in culture, have not been developed, and the specific molecular and cellular features of LAM cells that drive the pathogenesis of LAM have remained elusive. To this point, the establishment of human lymphangioleiomyomatosis cell models have been attempted from LAM patients, after biopsy or transplant, grown as a mixture of TSC2 wild-type and TSC2-null cells, with increased activation of mTOR [35,36,37], but the pure clonal population of TSC2-null pulmonary cells has not been established. An attempt to reprogram LAM lung cells were developed by the induced pluripotent stem cell (iPSC) lines that demonstrated normal TSC2 and TSC1 expression, suggesting that TSC2 deficiency inhibited the production of iPSC lines LAM iPSC, as defined by TSC2 mutation, but do not seem to grow as a clonal population in cell culture [38]. In 2020, Guo M and his colleagues developed a novel single-cell and single-nuclei RNA sequencing to analyze the LAM cells from human lungs and uterus tissue and identify LAM^CORE^ cells [39] but did not in cell culture. In our study, more LAM cell clusters were observed in the diameter sizes (70 μm and 100 μm) by the HMB45-positive immunohistochemical analysis. We then established a unique method using two different sizes of cell strainer to obtain LAM cell clusters (size between 100 μm to 70 μm) from patients’ lung tissue and culture the cells with SmGM^TM^- 2 smooth muscle cell growth medium (Lonaz). The LAM cells were studied at the early passage 1–3. Characterization of LAM cells was carried out at the first passage using immunocytochemical staining HMB45. However, the limitation in this LAM cell culture model is that there is no perfect control of the human cell line to compare with isolated LAM cells in the protein level of HMB45 or TSCs to evaluate differentiated features in this culture system.

The previous studies suggest that the metastatic spread or migration of LAM cells with positive reactivity with monoclonal antibody HMB45 plays an essential role in the pathogenesis of LAM [40,41]. Patients with sporadic LAM exhibit inactive mutations of TSC1 or TSC2, which activate the mTOR signaling pathway and increased cell proliferation and migration [23,34]. mTOR has been reported to regulate the LIMK and cofilin signaling pathways [23]. The phosphorylation of cofilin, an actin-cleaving protein, determines the direction of cell migration [32] and cancer cell metastasis [34,42]. Moreover, we showed overexpression of mTOR and HMB45 in the lungs of LAM patients. Sirolimus revealed efficacy in patients with LAM, as it inhibited the expression of mTOR and p-cofilin and reduced the interstitial LAM septal thickness in the lung tissue. The HMB45 level, which represents the total number of LAM cells, is significantly decreased in patients with LAM treated with sirolimus. This finding is consistent with a previous study by Cai X et al. [43], which reported that patients receiving sirolimus had a progressive loss of circulating LAM cells [43]. Taken together, the decreased p-cofilin levels determined by Western blots and the lower expression at the edge of the interstitial LAM septum determined by confocal microscopy suggest that the decrease in LAM cells in the peripheral blood may result from the clearance of LAM cells in the lungs and a reduced ability of the cells to migrate from the interstitial LAM septum into the circulation.

The reason why progressive loss of LAM cells occurs in the lungs after sirolimus treatment without decreases in cyst size or number is not well understood. Indeed, our specimens revealed that infiltrating LAM cells caused destruction of the lung parenchyma. The slow clearance of LAM cells in the lungs does not help to repair the alveolar septa and subsequently leaves stable cysts of variable size. In addition, the fact that only the LAM septal thickness was significantly different after sirolimus therapy suggests that the nodules and cystic lesions represent later stages of LAM. Therefore, we propose that the formation of cystic lesions originates from LAM cell infiltration of the septum, which shows the best response to sirolimus therapy, and when cysts and nodules are present, they represent an irreversible morphological stage of this disease. Our findings warrant further exploration, and if available, sequential wedge biopsies from LAM patients during sirolimus therapy may provide more evidence. There are so many previous reports that mTOR inhibitor reduced the proliferation and migration ability. P-cofilin is less in the treated group than the non-treated one. Still, there are two hypotheses: (1) decrease of p-cofilin is associated with the inhibition of the proliferation and migration ability; (2) decrease of p-cofilin is only a marker of treatment. To prove the hypothesis that sirolimus inhibits LAM activity through phosphorylation of cofilin, in this study, we show that sirolimus inhibited the phosphorylated level of cofilin. Future studies should focus on using the inhibitors for phospho-cofilin levels and cofilin siRNA to assess the cell signal mechanism in the LAM cells. Moreover, we suspect that the volume of LAM cells may be reduced by sirolimus, but we have no suitable equipment to measure the volume of LAM cells. The “volume” is a three-dimensional (3D) shape. However, this study’s limitation is applying immunohistochemistry and catching an image (2D image) to measure the cyst area and septum thickness.

Our study is the first report to perform histological structural analysis in LAM patients treated with and without sirolimus. Sirolimus effectively reduced the expression of p-cofilin and interstitial septal thickness, which may be a novel mechanism by which sirolimus protects against cystic destruction in patients with LAM.

## 4. Materials and Methods

### 4.1. Characteristics of Patient Lung Tissue

We obtained the human residual lung tissues from patients without LAM (non-tumor area of lung cancer surgical specimen) and patients with LAM undergoing bilateral sequential lung transplantation (BSLT) at National Taiwan University Hospital. [29]. The clinical characteristics of the patients are shown in Table 1. The study protocol for tissue donation was approved by the Human Research Ethics Committee of National Taiwan University Hospital (Institutional Review Board 201911023RINB) and conducted in concordance with the principles of the Declaration of Helsinki. Written informed consent was obtained from each patient. The number of cyst area and thickness of the LAM septum were utilized to characterize LAM cells by HMB45 immunohistochemistry staining. At least 3–5 slides were evaluated from every patient. In total, we had 7 LAM patients (total 188 septa counts, 93 cyst counts) vs. 6 LAM patients with sirolimus (total 128 septa counts, 72 cyst counts). Frozen tissue samples from 3 control patients with non-LAM diseases were acquired to compare their protein expression levels with those of 7 patients with LAM (3 patients with LAM and 4 patients with LAM treated with sirolimus).

### 4.2. Immunohistochemical Analysis

Immunohistochemical analysis of lung tissues was performed with primary antibodies against HMB45 (Santa Cruz Biotechnology, SC-59305, Dallas, TX, USA), desmin (Thermo Fisher, RB-9014-P, Waltham, MA, USA) and p-cofilin (Cell Signaling, 8354, Danvers, MA, USA). LAM cells were stained with HMB45 [24] to assess nodules, cystic area, and interstitial septal thickening. To evaluate protein expression, lung tissue sections were incubated with rabbit anti-p-cofilin or anti-desmin and mouse anti-HMB45 antibodies, followed by incubation with an Alexa-488-conjugated secondary antibody (green, Invitrogen, Waltham, MA, USA) for p-cofilin or a Cy3-conjugated secondary antibody (red, Invitrogen, Waltham, MA, USA) for HMB45 and observation with a confocal microscope (Confocal TCS SP8XL; Leica, Wetzlar and Mannheim, Germany) at the Microscope Core Laboratory of Chang Gung Memorial Hospital.

### 4.3. Assessment of Cystic Area, and Interstitial Septal Thickness

The number of nodules, cyst area, and thickness of the LAM septa were utilized to characterize LAM cells by HMB45 staining. Under microscopic examination with an ImageXpress Micro Confocal system (Molecular Devices), nodules, cystic areas, and interstitial septal thickness were defined as HMB45 positive, as calculated using the “NIS Elements” imaging software from Nikon. The number of cyst area and thickness of the LAM septa were utilized to characterize LAM cells by HMB45 staining. We used at least three tissue slides. From each slide, we selected the cysts, and thickness of the septa in HMB45 positive. In total, we had 7 LAM patients, (total 188 septa counts, 93 cyst counts) vs. 6 LAM patients (total 128 septa counts, 72 cyst counts).

### 4.4. Isolation and Culture of LAM Cells

In the HMB45-positive immunohistochemical analysis, more LAM cell clusters were observed the diameter size between 100 μm to 70 μm. We then established a novel method to obtain LAM cell clusters and culture the cells. The diameter of the lung tissue was cut into small pieces (1cm^3^) and collected in a 50 mL tube, then the tube was gently shaken for 5 min at 50 Hz in culture medium Dulbecco’s modified Eagle medium–F12 (Gibco, Rockville, MD, USA), to separate the LAM nodule. After that, the supernatant fraction (approximately 8 mL) was transferred and filtered through a 100 μm cell strainer (BD Falcon, 352360, Franklin Lakes, NJ, USA) in to a new 50 mL tube. Then, the supernatant fraction in the 50 mL tube was repeat to filter through a 70 μm cell strainer (BD Falcon, 352340, Franklin Lakes, NJ, USA). After the 100 μm to 70 μm filter process, the LAM cell clusters (the size between 100 μm to 70 μm) were collected from the 70 μm cell strainer. The LAM cell clusters were resuspended and 10mL of proliferating medium (SmGM- 2 smooth muscle cell growth medium) were added containing 5% fetal bovine serum (FBS), 1 μg/mL hydrocortisone, 10 ng/mL human epidermal growth factor, 3 ng/mL basic fibroblast growth factor, and 10 μg/mL heparin (Lonza, Walkersville, MD, USA) in 10 cm plates. LAM cell clusters were incubated at 37 °C in 5% CO2–95% air (Figure 5a). After 24 h, the medium was changed and thereafter every 2–3 days. The LAM cells were studied at the early passage 1–3. Characterization of LAM cells was carried out at the first passage using immunocytochemical staining HMB45 (Santa Cruz Biotechnology, SC-59305, Dallas, TX, USA) and 90% positive reactivity with HMB45 antibody.

### 4.5. Western Blot Analysis Characteristics of Patient Lung Tissue

For Western blotting, immunoblotting was performed using anti-HMB45 (Santa Cruz Biotechnology, SC-59305, Dallas, TX, USA), anti-total mTOR (EnoGene, Ab-2481, New York, NY, USA), and anti-p-cofilin (Cell Signaling, 8354, Danvers, MA, USA) as primary antibodies. Secondary antibodies specific for peroxidase-conjugated anti-mouse IgG or anti-rabbit IgG (Jackson ImmunoResearch, West Grove, PA, USA) were used as needed. The blots were visualized using an enhanced chemiluminescence detection system (Amersham, Piscataway, NJ, USA) and samples were normalized to glyceraldehyde 3-phosphate dehydrogenase (GAPDH) (Santa Cruz Biotechology, SC-32233, Dallas, TX, USA) and quantified by densitometry.

### 4.6. Cell Viability and Proliferation Assay

Cell viability was assessed with 3-[4,5-dimethylthiazol-2-yl]-2,5-diphenyltetrazolium bromide (MTT)(Sigma-Aldrich, Burlington, MA, USA) assay according to the manufacturer’s instructions, the absorbance was measured at a 570-nm wavelength using a microplate (ELISA) reader. The proliferative activity of LAM cells and the effects of sirolimus were determined using an ELISA-based 5-bromo-2-deoxyuridine (BrdU) incorporation assay kit (Roche Diagnostics Co., Basel, Switzerland) according to the manufacturer’s instructions.

### 4.7. Cell Migration Assay

A transwell filter chamber (Corning Costar, Corning, NY, USA) with an 8.0 μm pore size was used to evaluate the effect of sirolimus on migration. Human LAM cells were seeded at a density of 5 × 105 cells per well and pretreated with sirolimus for 2 h. To initiate the chemotaxis assay, cells in 200 μL of DMEM without FBS and growth factors were added to the upper chamber, and the bottom chamber was filled with 600 μL of cell culture medium as a chemotactic factor for cell movement. LAM cells were supplemented with or without the sirolimus (0.1 μM) and allowed to migrate for 4 h. The cells on the filter membrane were stained with Liu’s stain according to the manufacturer’s instructions [44].

### 4.8. Statistical Analysis

The mean and standard error of the mean (SEM) were used to describe the data. Differences between two groups were determined by unpaired *t*-tests. One-way ANOVA and the post hoc Bonferroni test were used to compare data among multiple groups. A value of *p* ≤ 0.05 was considered statistically significant.

## Figures and Tables

**Table 1:** Study population characteristics.

	LAM	LAM/Sirolimus
Subjects *n*	7	6
Male/Female	0/7	0/6
Recipient age at listing (years)	48.43 (38.21–55.75)	45.17 (37.01–53.33)
Recipient age at transplant (years)	48.43 (41.11–55.75)	46.50 (37.95–55.05)
FEV1 (% predicted)	29.83 (7.49–52.17)	21.70 (14.73–28.67)
FEV1/FVC	40.66 (21.69–59.62)	33.85 (29.62–38.08)
DLco (ml/min/mmHg)	5.14 (4.50–5.77)	3.88 (1.47–6.29)
mPAP (mmHg)	22.43 (16.16–28.70)	28.40 (21.10–35.70)
PVR (Wood Unit)	2.31 (1.17–3.45)	3.22 (2.01–4.43)
CO (L/min/m^2^)	4.24 (3.74–4.74)	4.72 (3.81–5.63)
6MWD (m)	280.8 (137.02–424.58)	291 (171.75–410.25)
Septum (μm)	502.91 (457.63–548.19)	278.83 (212.5–345.15) ***

LAM: lymphangioleiomyomatosis; FEV1: forced expiratory volume in 1 s; FVC: forced vital capacity; DLco: diffusing capacity of the lung for carbon monoxide; mPAP: mean pulmonary arterial pressure; PVR: pulmonary vascular resistance; CO: cardiac output; 6MWD: 6-min walking distance. *** *p* < 0.001 compared to patients treated without sirolimus.

## Data Availability

The data presented in this study are available in article.

## Abbreviations

BSLT	bilateral sequential lung transplantation
CT	computed tomography
ECMO	extracorporeal membrane oxygenation
GAPDH	glyceraldehyde 3-phosphate dehydrogenase
HRCT	high-resolution computed tomography
ILD	interstitial lung disease
LAM	lymphangioleiomyomatosis
LIMK	LIM kinase
LT	lung transplantation
mTOR	mammalian target of rapamycin
mTORC1	the mammalian target of rapamycin complex 1
PKA	protein kinase A
Rac1	Ras-related C3 botulinum toxin substrate 1
Rheb	Ras homolog enriched in brain
SEM	standard error of the mean
SLT	Single LT
SSh1	Slingshot-1
TESK1	Testis-specific kinase 1
TORSC	Taiwan Organ Registry and Sharing Center
TSC	tuberous sclerosis complex
TSC1	Hamartin
TSC2	tuberin
VEGF-D	vascular endothelial growth factor-D
